# Metagenomes of rectal swabs in larger, advanced stage cervical cancers have enhanced mucus degrading functionalities and distinct taxonomic structure

**DOI:** 10.1186/s12885-022-09997-0

**Published:** 2022-09-01

**Authors:** Tatiana V. Karpinets, Xiaogang Wu, Travis Solley, Molly B. El Alam, Travis T. Sims, Kyoko Yoshida-Court, Erica Lynn, Mustapha Ahmed-Kaddar, Greyson Biegert, Jingyan Yue, Xingzhi Song, Huandong Sun, Joseph F. Petrosino, Melissa P. Mezzari, Pablo Okhuysen, Patricia J. Eifel, Anuja Jhingran, Lilie L. Lin, Kathleen M. Schmeler, Lois Ramondetta, Nadim Ajami, Robert R. Jenq, Andrew Futreal, Jianhua Zhang, Ann H. Klopp, Lauren E. Colbert

**Affiliations:** 1 Department of Genomic Medicine, The University of Texas MD Anderson Cancer Center, Houston, TX USA; 2 Department of Radiation Oncology, The University of Texas MD Anderson Cancer Center, Houston, TX USA; 3 Department of Gynecologic Oncology, The University of Texas MD Anderson Cancer Center, Houston, TX USA; 4 Alkek Center for Metagenomics and Microbiome Research, Baylor College of Medicine, Houston, TX USA; 5 Department of Infectious Diseases, The University of Texas MD Anderson Cancer Center, Houston, TX USA; 6 Program for Innovative Microbiome and Translational Research, Department of Genomic Medicine, The University of Texas MD Anderson Cancer Center, Houston, TX USA

**Keywords:** Metagenomics, Mucus layer, Gut, Cervical cancer, Tumor size, Bacteria, *Bacteroidales*, *Clostridiales*

## Abstract

**Background:**

Gut microbiome community composition differs between cervical cancer (CC) patients and healthy controls, and increased gut diversity is associated with improved outcomes after treatment. We proposed that functions of specific microbial species adjoining the mucus layer may directly impact the biology of CC.

**Method:**

Metagenomes of rectal swabs in 41 CC patients were examined by whole-genome shotgun sequencing to link taxonomic structures, molecular functions, and metabolic pathway to patient’s clinical characteristics.

**Results:**

Significant association of molecular functions encoded by the metagenomes was found with initial tumor size and stage. Profiling of the molecular function abundances and their distributions identified 2 microbial communities co-existing in each metagenome but having distinct metabolism and taxonomic structures. Community A (*Clostridia* and *Proteobacteria* predominant) was characterized by high activity of pathways involved in stress response, mucus glycan degradation and utilization of degradation byproducts. This community was prevalent in patients with larger, advanced stage tumors. Conversely, community B (*Bacteroidia* predominant) was characterized by fast growth, active oxidative phosphorylation, and production of vitamins. This community was prevalent in patients with smaller, early-stage tumors.

**Conclusions:**

In this study, enrichment of mucus degrading microbial communities in rectal metagenomes of CC patients was associated with larger, more advanced stage tumors.

**Supplementary Information:**

The online version contains supplementary material available at 10.1186/s12885-022-09997-0.

## Background

Cervical Cancer (CC) is the fourth most common cancer in women worldwide [1]. The vast majority of these cases are caused by the human papillomavirus (HPV). Most individuals infected by the virus do not develop the cancer, as the viral infection is cleared before the virus integrates in the genome [2]. Prior reports [3] have identified connections between the local (cervical/vaginal) microbiome and HPV infection and between cervical intraepithelial neoplasia and CC development. We previously reported differences between CC patients and healthy controls [4] and associations of gut diversity and composition with survival of CC patients based on 16Sv4 rRNA sequencing [5]. The effect of gut microbial functions on development and progression of CC in human is unknown. In a murine model of HPV cancer, we recently identified a significant association between gut and vaginal microbiomes and impact of the association on the development and progression of CC [6]. Causal relationships are well established between the gut microbiome composition and the development of other cancers, such as colon cancer [7]. Additionally, a growing body of evidence links microorganisms to the efficacy of cancer therapies [8–11].

In most studies, bacterial DNA extracted from fecal samples is used as proxy for evaluation of the gut microbiome composition. However, the microbial communities adjoining and populating the outer mucus layer of the intestine are different from stool as a whole [12] and may be more relevant in modulating immune function. Indeed, composition of bacteria within the stool primarily reflect the dietary habits of patients, while microbial communities adjoining the mucus layer [13, 14] also protect the gut epithelial surface that holds many important immune and metabolic functions. The difference may be more pronounced when the mucus layer is somehow disturbed because of a disease [15].

The mucus layer consists of an outer mucus layer, which is a habitat for commensal bacteria, and a smaller inner layer, which is attached to the epithelial cells and is lacking commensal bacteria [16]. This protective coatings are the crucial interface between the host and microorganisms [17] and are mainly (~ 89%) comprised of glycans (polysaccharides) attached by O-glycosylation to MUC2 (mucin 2) protein [18]. Galactose and N-acetylgalactosamine are major components of mucus glycan in normal human descending colon, although the structure varies in other parts of the intestine [19]. The mucus glycans provide attachment sites for bacteria [20] and select species that can influence the mucus layer structure and the host [21] by degrading mucin glycans, metabolizing products of the degradation, and producing metabolites that affect the host, positively or negatively. According to recent studies [22, 23], mucins have also potent beneficial properties and can modulate microbial phenotypes suppressing quorum sensing, biofilm formation, and secretion of toxins. Thus, the microbial community adjoining and populating the outer mucus layer occupies a rather distinct environment within the intestine and can be affected not only by diet, but also by the mucus barrier and by the host.

Previous bacterial 16S rRNA gene sequencing studies found that stool and rectal swab microbiotas from the same subject were similar and that rectal swabs can be used as a proxy for fecal samples [13, 14, 24]; However, in light of the findings referenced above, the mucus layer of the intestine, including rectum, can significantly modify phenotype of the adjoining microbial community. This means that even if the structure of the community is similar between stool and swabs, metabolic functions implemented by the same organisms in these different environments may be different. In addition, the use of Whole Genome Shotgun sequencing *(*WGS*)* instead of 16S RNA can provide finer resolution of the microbial community structure and functions and can reveal features not found by 16S.

In this study, we propose that molecular functions encoded by metagenomes, further referred as MA (Mucus Associated) because they populate the mucus layer or adjoin to it, are different among CC patients and are likely associated with their clinical characteristics. We therefore explore metabolic characteristics of microbial communities populating the mucus layer in rectums of 41 CC patients. Rectal swabs were used to sample the communities and then to sequence them by WGS.

## Methods

### Patient samples collection and processing

Forty-one patients treated at the University of Texas, M.D. Anderson Cancer Center or the Lyndon B. Johnson Clinic (LBJ) at Harris Health with a diagnosis of CC participated in the study. The patients were enrolled on an Institutional Review Board (IRB) approved prospective protocol. Informed consent was obtained to collect rectal swabs. All patient samples were acquired prior to receiving any treatment.

Samples were collected from each out of 41 patients by a clinician performing rectal exams using a matrix-designed quick-release Isohelix swab. The swabs were initially stored in 20ul of proteinase K and 400 μl of lysis buffer (Isohelix) within 1 h of sample collection and then were frozen and kept at -80 °C.

### WGS sequencing and metagenome assembly

Whole Genome Shotgun sequencing was performed on genomic bacterial DNA (gDNA), which was extracted to maximize bacterial DNA yield from specimens while keeping background amplification to a minimum [25, 26]. Libraries were constructed from each sample using the KAPA Hyper Prep Kit (Kapa Biosystems, Wilmington, MA, USA) and sequenced using the Illumina HiSeqX platform with the 2 × 150 bp paired-end read protocol. Sequencing reads were derived from raw BCL files which were retrieved from the sequencer and called into fastqs by Casava v1.8.3 (Illumina). The appropriate read preparation steps, such as quality control, trimming and filtering, and host DNA removal prior to further analysis, were performed using an in-house pipeline (Additional file 1: Figure S1; Additional file 2: Table S1). Briefly, paired-end raw sequence reads were filtered and trimmed using BBMap [27]. The trimmed reads were mapped to a hg38 reference database (GCA_000001405.28) using bowtie2 [28] to remove host contamination. The cleaned reads were then assembled to longer sequences (contigs) using both MEGAHIT [29] and metaSPAdes [30]. The assembled contigs were filtered, to remove those that were smaller than 1000 bp, and binned using MetaBAT2 [31]. The assembled and binned contigs were used for gene predictions by Prodigal [32]. Annotation of the genes by KEGG ortholog groups (KOs) was implemented by KofamKOALA [33], and taxonomic classification of the contigs was done by CAT and BAT [34]. The read coverage of each assembled contig was calculated by aligning the cleaned reads directly to the contig using BBMap and by counting the mapped read by featureCounts [35]. The read coverage, GC content, taxonomic and functional annotations for each gene/contig were summarized using a Perl script. All the software tools were running with default parameters if not specified. Versions and sources of the software tools or packages used in the pipeline are listed in Additional file 2: Table S1. Output of the assembly pipeline was a set of assembled contigs for each sample, their taxonomic annotation and read coverage, gene predictions for each contig, and functional annotation of each gene by KEGG Orthologous Group (KO) if found.

### Computational analysis of assembled genomes

Annotations of assembled contigs for all samples were aggregated into 1 table, referred to as the Metagenome Function Abundance (MFA) table (Additional file 1: Figure S1b, S2). Each column in the table represents a metagenome, and each row represents a predicted known molecular function annotated by KO. Thus, each cell in the MFA table has a quantity of the specified (KO id) molecular function in the specified sample (Sample id). Quantification of the molecular functions by MFA table is explained using a toy example provided in Additional file 1: Figure S2. The MFA table was normalized using total number of reads in each sample and then multiplied by 1,000,000. Further analysis of the normalized MFA table included unsupervised and supervised methods, annotation by biological processes and pathways, and integration with clinicopathological characteristics of the patients (Additional file 1: Figure S1c).

#### Unsupervised analysis of MFA table

The MFA table was filtered to select KOs found in at least 30 patients, log2-transformed and centered using the median, and then hierarchically clustered by the open source clustering software [36] with default parameters. The inferred clusters of samples were tested for association with clinical information including age, CC stage and tumor size, using fisher.test() and wilcox.test() functions in R. The inferred clusters of KOs were searched for overlap with known KEGG referenced pathways and modules using the “Search&Color Pathway” tool in KEGG mapper [37, 38] with further manual curation of the results.

#### Supervised analysis of MFA table

The analysis was used to find differentially abundant KOs in MA metagenomes of patients with large versus small tumors. Samples for the analysis were selected by sorting all 41 samples by tumor size of the CC patients and assigning top 14 samples with largest tumors to LT-group and bottom 14 samples with smallest tumors to ST-group for the comparison. All KOs found in at least 1 sample were included in the analysis. Fisher’s test and Mann Whitney tests were used to find differentially abundant KOs between the group with *p-*value < 0.05 (either test) without adjustment. The KO was considered enriched in LT-group if Fisher’s test *p-*values < 0.05 and the KO is more common in the group. If the Fisher’s test *p-*value > 0.05 but the Wilcoxon test *p-*value < 0.05 than the enrichment was inferred by difference in mean abundances between ST- and LT-groups. The differentially abundant KOs were searched for overlap with known KEGG referenced pathways and modules as described above. The overlapping KOs were used to infer the pathways enrichment score calculated as ratio of difference between percentage of KOs overlapped with the pathway in ST- and LT-groups to sum of the percentages. Only top scored pathways that include 9 and more KOs for LT-group and 4 and more KOs for ST-group are considered.

Annotation of the differentially abundant KOs by carbohydrate-active enzymes (CAZy) families was implemented using the mapping table between the KO ID and CAZy family ID [39]. The table was downloaded from KEGG on 26 October 2019.

The differentially abundant KOs were further used to quantify relative abundances of microbial subpopulations encoding the functions in ST and in LT group of samples. The decomposition of cell populations, referred to as ST-dominant and LT-dominant, was implemented based on characteristics of density plots of KOs associated with each subpopulation. Namely, the differentially abundant KOs in ST and LT group were used to construct 2 normal curves for ST-abundant and LT-abundant KOs. It was assumed that parameters of the curves, mean and standard deviation, characterize molecular functions expressed by the microbial community dominating in either ST-group or LT-group, although each of the groups is comprised of both communities. To quantify abundances of the communities in each group, we fitted the 2 normal curves to density plots of all KOs found in the two groups using multiple linear regression. The obtained fitting coefficients were used to quantify the dominance of each community in metagenomes.

### Survival analysis

The analysis was used to associate the Recurrence Free Survival (RFS) probability with clinicopathological characteristics and KEGG pathway [40]. Activity *of each* pathway found to be associated with the tumor size was quantified for each patient using the mean value of log2-transformed normalized abundances of KOs involved in the pathway. The mean value is referred to as the pathway activity score. To generate Kaplan–Meier plots for the pathway activity score, we categorized the score based on a cut-off set at the first quartile. Observations for each variable falling within the first quarter were labeled as “Low” and those greater than the first quartile cut-off were labelled as “High”. The analysis was implemented for each pathway identified as differentially enriched in either small or large tumor group metagenomes. We used WHO standards to set cut-offs to categorize BMI into “Underweight/Normal weight” vs. “Overweight/Obese”. We used a cut-off set at the median for other continuous clinicopathological characteristics, namely, age and tumor size. Cox proportional hazards regression analysis [41] was used to evaluate predictive value of KEGG pathways and clinical characteristics on RFS time. The R packages ‘survival’ and ‘survminer’ were used to compute the survival curves, and to visualize them as Kaplan–Meier plots.

### Taxonomic characterization of the cohort

All predicted contigs annotated by Length (L), Depths (D), and by taxonomy were used to quantify abundances of the taxa. For each sample, depth of the contig was multiplied by its length, and then the values were summarized for all contigs that belong to the same taxa to quantify the taxa abundance.

The values were normalized using the sum of the values for all contigs identified in the sample.

The OTU (operating taxonomic units) table was created by merging the abundances of all identified taxa for all 28 samples. Two approaches were used to find differentially abundant putative taxa between LT and ST group. In the first approach, the OTUs were selected by Fisher’s exact test (for rare OTUs) and Mann Whitney tests (for common OTUs) with *p-*value cutoff < 0.05 (either test) without adjustment. In the other approach, the Linear discriminant analysis (LDA) Effect Size (LEfSe) [42] available as a Galaxy [43] module was used to determine the taxa that are differentially abundant between LT and ST groups. The analysis was run with default parameters, except the threshold for the logarithmic LDA score for discriminative taxa. The threshold was set to 2.5 instead of 2.

To reveal taxa involved in glycan degradation pathway in each metagenome we have selected all contigs that encode enzymes involved in the pathway. The set of unique taxa from taxonomic annotation of the contigs was considered as the representative taxa of the pathway. To create an OTU table of the representative taxa, each OTU were quantified by multiplying the length and the depth of each selected contig and by summarizing the obtained values for all contigs encoding the OTU. The 100% stacked area plot was used to visualize taxonomic structure of the community involved in the pathway across all studied samples. The Pearson correlation coefficients were used to evaluate association of the log2-transformed score of the KEGG pathway with the abundance of each identified taxon.

## Results

### Study cohort

Patients were enrolled on an IRB approved (MDACC 2014–0543) study for longitudinal sampling of the gut microbiome (Additional File 2: Table S2). For all 41 patients enrolled on the study, median age was 49 (range 29 to 72 years), and median BMI was 28.6 (range 17.5 to 46.7). Most patients were Non-Hispanic White (44%) or Hispanic (44%) and had squamous carcinoma (74%) and stage II disease (54%). Median tumor size was 5.4 cm (range 1.8 cm to 11.5 cm). More information on clinicopathological characteristics of the patients is provided in Additional File 2: Table S2.

**﻿**

### Functional richness of MA metagenome associates with larger tumor size and with advanced CC stage

**Fig. 1:**
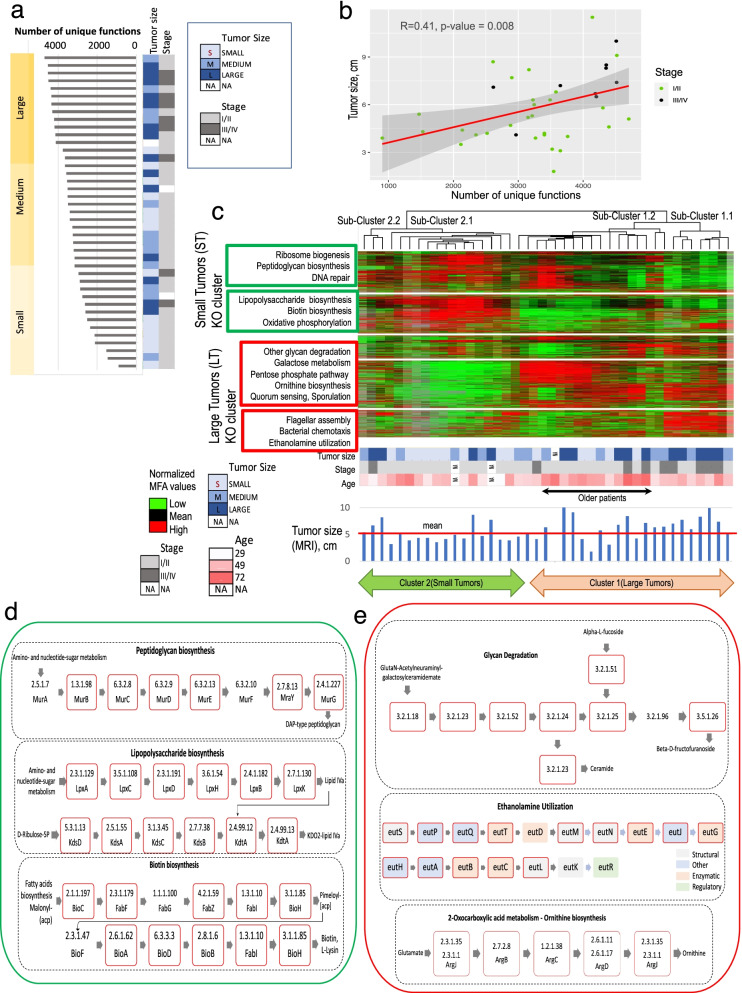
Association of molecular functions of MA metagenomes with KEGG pathways and clinical characteristics of CC patients. **a** Large number of unique functions is associated with larger tumor size and advance CC stage. **b** Linear relationship between the number of unique functions and the tumor size. **c** Unsupervised hierarchical clustering of metagenomes in terms of abundances of molecular functions. The heat map of the clustering shows abundances of 2396 KOs as rows and 41 MA metagenomes as columns. Boxes with enriched KEGG pathways are colored by red if the clustered molecular functions are more abundant in metagenomes of patients with Large tumors (Cluster 1) and in green if the molecular functions are more abundant in metagenomes of patients with Small tumors (Cluster 2). **d** Synthesis of peptidoglycan, lipopolysaccharides and biotin are enriched among KOs that are abundant in Cluster 2 (Small tumor) metagenomes. **e** Glycan degradation, ethanolamine utilization and production of ornithine are enriched among KOs that are abundant in Cluster 1 (Large tumor) metagenomes. Enzymes involved in the enriched pathways have red borders if they are found among KOs of the Cluster 1 or Cluster 2

Among clinicopathological characteristics, the tumor size and CC cancer stage had the most significant associations with total number of predicted genes and functions (Table S3, Fig. 1a, b). Greater number of unique KEGG orthologous groups (KOs) was significantly associated with advanced stages (III/IV) of cervical cancer (*P* = 0.005; Fig. 1a) and with larger tumor size (*P* = 0.02; Fig. 1a), which had linear correlation with the number of unique functions (*R* = 0.41, *P* = 0.008) (Fig. 1b). No significant association of the characteristic (*P* = 0.21) was found with BMI that may indirectly characterize eating and dietary habits of the patients.

### Unsupervised hierarchical clustering reveals microbial communities with 2 distinct profiles of molecular functions

Next, we explored whether specific genes or functions were associated with clinicopathological characteristics using unsupervised hierarchical clustering of KO abundances. We selected 2,396 KOs that were found in greater than 30 samples for the analysis. These KOs clustered tumors into 2 large clusters and 4 sub-clusters (Fig. 1c). In general, Cluster 2 was comprised of smaller tumors (“ST cluster”), when compared with Cluster 1 (“LT cluster”; t-test *P* = 0.06). Within the LT cluster, Sub-cluster 1.1 was dominated by younger patients (t-test *P* = 0.05) with large tumors (t-test *P* = 0.03).

The KEGG pathway analysis of the ST cluster and the LT cluster using KEGG mapper [38] identified a set of pathways enriched in each of the clusters (Fig. 1c). The ST Cluster was enriched with pathways associated with rapid microbial cell proliferation, including ribosome biogenesis (Additional File 1: Figure S3a), DNA repair (Additional File 1: Figure S3b), oxidative phosphorylation (Additional File 1: Figure S3c), and synthesis of peptidoglycan and lipopolysaccharides **(**Fig. 1d). Almost all enzymes involved in synthesis of biotin (vitamin B7) were also found in the ST cluster **(**Fig. 1d).

The LT cluster (Cluster 1 in Fig. 1c) was enriched with pathways associated with bacterial stress response, indicated by activation of quorum sensing (Additional File 1: Figure S4), sporulation, and degradation of glycan. The glycan degradation pathways included high activity of the KEGG pathway, “Other glycan degradation” (Fig. 1e). In addition, pathways involved in utilization of glycan degradation byproducts, such as fructose and mannose (Additional File 1: Figure S5a), and especially galactose (Additional File 1: Figure S5b), the major constituent (~ 85%) of glycans in normal human gastric mucus [44] were enriched in the LT cluster. Both branches of the pentose phosphate pathway, the oxidative branch maintaining redox balance in stress conditions and non-oxidative branch that supply glycolysis with intermediates derived from pentoses [45], were also more active in the LT cluster (Additional File 1: Figure S6). Most enzymes involved in utilization of ethanolamine (Fig. 1e), a breakdown product of human and bacterial cell membrane [46], and enzymes involved in production of ornithine (Fig. 1e), a precursor for synthesis of polyamines, such as putrescine [47, 48], were found enriched in the LT cluster. The biological processes of flagellar assembly and chemotaxis (Additional File 1: Figure S7) were also u*p-*regulated in the LT cluster, revealing the importance of mobility for bacteria residing in the metagenomes [49].

### Supervised comparison of metagenomes associated with largest and smallest tumors

**Fig. 2:**
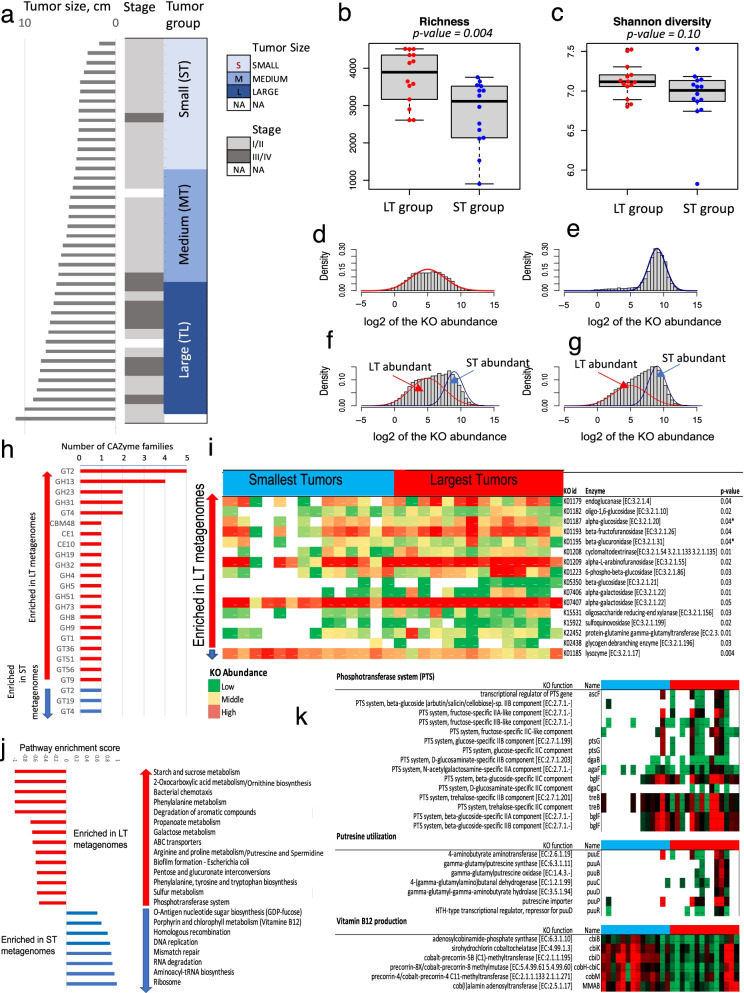
Supervised comparison of Largest tumors (LT-group) and Smallest tumors (ST-group) of MA metagenomes. **a** Grouping of samples into ST- and LT-groups for supervised comparison. **b**,**c** Greater richness and diversity of molecular functions in LT-group of metagenomes. **d**,**e** Density plots of differentially abundant KOs in LT- (**d**) and ST- (**e**) groups of metagenomes. Parameters of the normal distributions were used to find amplitudes of LT- and ST-abundant molecular functions among all KOs in each tumor group. **f**,**g** Density plots of all KOs identified in LT- (**f**) and ST- (**g**) groups of metagenomes. **h** Carbohydrate Active enZymes (CAZymes) identified in LT- and ST- groups. i Glycoside hydrolases involved in degradation of glycans (EC 3.2.1.-) are differentially enriched in LT- versus ST- group of metagenomes. **j** KEGG pathways enriched among KOs differentially abundant between ST- and LT- groups of metagenomes. **k** Some KEGG pathways differentially abundant between the groups. Phosphotransferase and putresine utilization pathway are more abundant in LT-group, while vitamin B12 production is more abundant in ST-group

To confirm the association of large tumor size with glycan degrading microbial communities, we divided patient samples into “Large Tumor” (LT-group) and “Small Tumor” (ST-group) groups. The LT-group included 14 metagenomes of patients with the largest tumors, and the ST-group had 14 metagenomes of patients with the smallest tumors (Fig. 2a). The LT-group were also significantly more enriched in patients with stage III/IV of CC then ST-group (chi-squared contingency table tests *p-*values 0.02), since tumor size is roughly correlated with stage in the 2009 FIGO staging system used in the study. The diversity and richness of molecular functions was higher in LT versus ST-group (Fig. 2b and c respectively). There were 1,496 differentially abundant KOs between the groups with most of the KOs (75%) were more abundant in the LT-group. The density plots of the differentially abundant KOs were also different between the groups. The KO abundances were significantly more scattered around the mean (standard deviation is 2.6) in LT-group when compared with ST-group (standard deviation is 1.3). The KO abundances mean value was almost twice as low in LT-group than in ST-group (Fig. 2d and e respectively). The observed density plots indicated that 2 distinct populations (sets) of molecular functions, referred to as LT-abundant and ST-abundant, might coincide in each metagenome but be most representative in either LT- or ST-group of samples. This conclusion was confirmed by density plots of all KOs found in LT-group (Fig. 2f) and in ST-group (Fig. 2g). By fitting parameters of the normal distributions from Fig. 2d and e to density plots in Fig. 2f and g using multiple linear regression, we found that although both, LT- and ST- abundant functions were present in LT- and ST-group of metagenomes, the fitting parameter of LT-abundant population was increased in LT-group by 1.2 and the fitting parameter of ST-abundant population was decreased by 1.5 in the group.

### CAZyme families associated with small and large tumor metagenomes

To associate the LT-abundant KO population with the glycan degradation we compared abundances of carbohydrate-active enzymes (CAZyme) families in this population and in the ST- abundant KO population using CAZy database [39]. The database classifies CAZymes according to their functions, such as synthesis of complex carbohydrates or their hydrolysis. Glycoside hydrolases GH, which are enzymes involved in hydrolysis of glycosidic bonds between carbohydrates or between a carbohydrate and a non-carbohydrate moiety, were highly enriched in LT abundant KO population (Fig. 2h). There were 16 GH among KOs in the population, and none in the ST abundant population, which included only 3 Glycosyl Transferases (GT); the enzymes that are mainly involved in biosynthesis of disaccharides, oligosaccharides, and polysaccharides. We further compared ST- and LT-abundant KO populations in terms of abundances of glycosidases, enzymes annotated by EC 3.2.1- involved in hydrolyzes of O- and S-glycosyl compounds comprised the mucus layer. There were 32 glycosidases in 28 samples, and many of them were significantly more abundant in metagenomes of patients with large tumors (Fig. 2i). Only lysozyme, a known component of two-component cell lysis cassette in bacteriophages [50], was found to be significantly more abundant in the small tumors group of patients.

### Biological processes associated with small and large tumors

The KEGG pathway analysis of differentially abundant KOs was further used to identify specific biological processes represented by LT-abundant and ST-abundant KOs (Fig. 2j). The representative pathways revealed by the analysis were consistent with results of the supervised analysis of 41 samples. Very active proliferation in ST-group of metagenomes were indicated by a high pathway enrichment score (ST- versus LT-abundant population) for DNA replication, ribosome, t-RNA biosynthesis, homologous recombination, and mismatch repair. Stress response and degradation of the mucus layer in LT-group were indicated by enrichment of biofilm formation pathway, galactose metabolism (Fig. 2j, Additional File 1: Figure S8), and by a significant increase in the abundance of trehalose-specific PTS components (Fig. 2k) among LT- versus ST-abundant KOs. Ornithine biosynthesis (Additional File 1: Figure S9), putrescine and spermidine metabolisms (Additional File 1: Figure S8; Fig. 2k) were also enriched in LT- abundant KOs, while enzymes involved in vitamin B12 production were enriched among ST-abundant KOs (Fig. 2k).

### Association of KEGG pathway with clinicopathological characteristics and recurrence free survival (RFS)

**Fig. 3:**
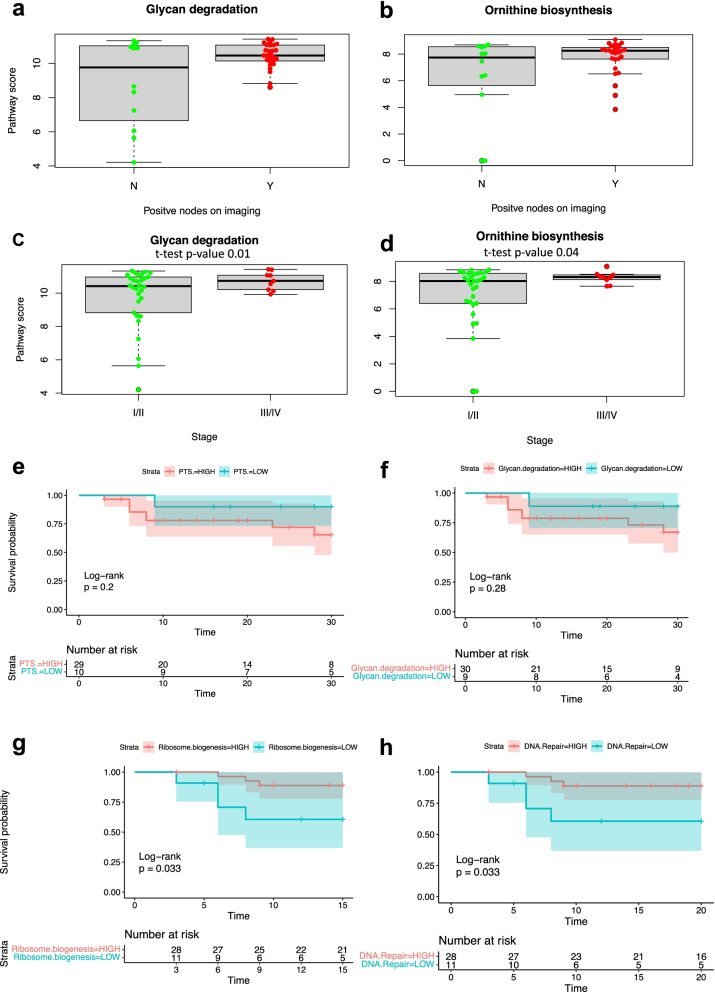
Association of KEGG pathway and clinicopathological characteristics. **a** Increased activity of Glycan degradation pathway and **b** Ornithine biosynthesis in metagenomes of patients with positive nodes on imaging. Categorization of patients into the negative (red color) and positive (green color) groups according to absence or presence of cancer cells in lymph nodes respectively revealed that 6 out of 12 patients with negative nodes had very low activity of Glycan degradation pathway. **c** Increased activity of Glycan degradation pathway and **d** Ornithine biosynthesis in metagenomes of patients with stage III/IV cervical cancer. **e**, **f** Negative association of Phosphotransferase system and of Glycan degradation pathway with recurrence free survival (RFS). Both pathways were enriched in metagenomes of patients with large tumors. The pathway activity was quantified in each metagenome by the mean value of log2-transformed normalized abundances of KOs involved in the pathway; the value is referred to as the pathway activity score, which was categorized as high in the first quartile and low in the rest. **g**, **h** Significant positive association of Ribosome biogenesis and of DNA repair pathways with RRS. Both pathways were enriched in metagenomes of patients with small tumors

None of the studied clinical characteristics showed significant (*p* < 0.05) association with RFS. This analysis was limited by the low number of events in the study cohort. Among KEGG pathways enriched in either LT- or ST-group of MA metagenomes, only a trend of negative association with RFS was found for high activity of glycan degradation pathway and of Phosphotransferase system (Fig. 3e and f). Both pathways were enriched in LT-group of MA metagenomes. Vice versa, significant positive association with RFS was found for high activity of Ribosome biogenesis and DNA repair pathways enriched in ST- group of MA metagenomes (Fig. 3g and h). Only a trend of negative associations with improved RFS (Additional File 1: Figure S10A and S10B) was observed for high stage tumors (log-rank *P* = 0.10) and for positive nodal status (log-rank *P* = 0.15), well known clinical characteristic of cancer progression and aggressiveness. Nonsignificant associations were likely because of the small number of patients in the study; very significant association of the same characteristics with survival was documented in large cohort of CC patients (> 10,000) [51]. Despite nonsignificant association with RFS, patients with positive nodes had significantly increased activity of glycan degradation pathway (*P* = 0.005) and ornithine biosynthesis (*P* = 0.05) in MA metagenomes (Fig. 3a and b). Activity of the pathways was also significantly higher in patients with stage III/IV tumors (Fig. 3c and d), while activity of ribosome biogenesis was higher in metagenomes of stage I/II patients (Additional File 1: Figure S11).

### Taxonomic associations with tumor size

**Fig. 4:**
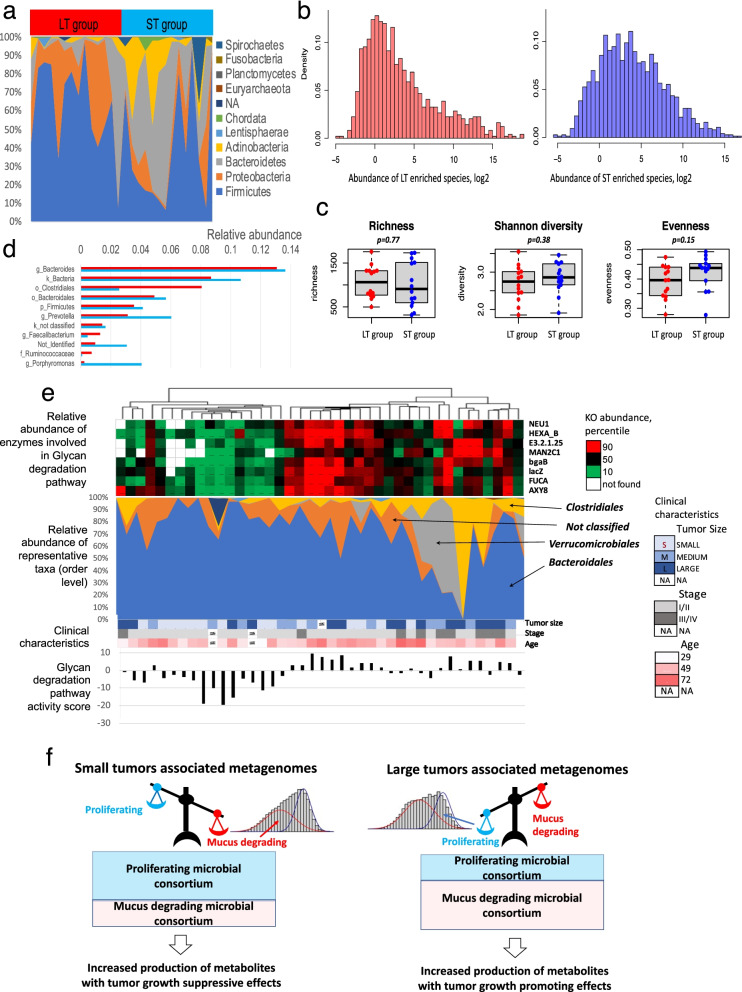
Taxonomic profiling and biological models of MA metagenomes in LT- and ST-groups. **a** Taxonomic structure of metagenomes at the *Phyla* level. **b** Density plots of differentially abundant species in LT- and ST-groups. Fisher’s test and Mann Whitney tests were used to find differentially abundant OTUs with *p-*value < 0.05 (either test) without adjustment. **c** Richness, diversity, and evenness of OTUs in LT and ST-groups. **d** Taxonomic annotation of most abundant contigs in LT- and ST-groups. **e **Glycan degradation pathway in terms of enzyme abundances (top) and representative taxonomic Order (bottom) of the enzymes in each patient. The clinical characteristics of each patient and the Glycan degradation pathway activity score are given at the bottom of the table. To reveal taxa involved in the glycan degradation pathway in each metagenome we have selected contigs that encode enzymes involved in the pathway and quantify abundances of each taxa considering length and read coverage of the contigs. The figure overlays abundances of enzymes with the relative abundances of taxa involved in glycan degradation across metagenomes. Samples (X axis) in the figure have the same order as in the unsupervised clustering of samples in terms of KOs in Fig 1c. f Biological models of Mucus Adjoining (MA) bacterial consortium in LT- and ST-groups

Difference in taxonomic structure of MA metagenomes in LT-group versus ST-group can be seen at different taxonomic levels (Fig. 4a, Additional File 1: Figure S12-S14) with dominance of Phylum *Firmicutes (*Class *Clostridia* and Order *Clostridiales)* and Phylum *Proteobacteria* in LT- group and Phylum *Bacteroidetes* (Class *Bacteroidia,* Families *Prevotellaceae* and *Ruminococcaceae* in ST-group. Like in case of differentially abundant molecular functions (Fig. 2d and e), the density plots of differentially abundant species were also different between the tumor groups (Fig. 4b) with a greater median abundance of species in ST-group. No difference, however, was seen between LT- and ST-groups of metagenomes in terms of species richness, diversity, and evenness (Fig. 4c).

Taxonomic annotation of most abundant contigs (OTUs) in each group and comparison of their abundances between groups revealed that many of the contigs belonged to Order *Bacteroidales,* and they were significantly more abundant in ST-group. There were 5 putative species of Genus *Porphyromonas (P. endodontalis, P.* sp *COT_239OH1446, P. bennonis, P.* sp *HMSC065F10 and P. uenonis*) and 2 species of Genus *Prevotella* (*P. Timonensis and P. Buccalis*) among *Bacteroidales* (Fig. 4d, Additional File 2: Table S4). None of the genera was significantly abundant in LT-group. Contigs annotated by Class *Clostiridia* including 12 putative species of Family *Ruminococcaceae* were more abundant in LT-group*.* Only 1 species of *Ruminococcaceae* (*AF41_9*) was found more abundant in ST group. The observations were consistent with results obtained by comparison of LT- and ST-groups using LEfSe (Additional File 1: Figure S15). The latter analysis has also identified less abundant taxa enriched in one of the groups, such as Class *Tissierellia,* Genera *Ezakiella, Murdochiella and Hungatella,* in ST-group, as well as Families *Ruminococcaceae, Lachnospieraceae, Enterobacteriaceae*, and Order *Enterobacterales* in LT group (Additional File 1: Figure S16).

### Taxonomic associations with glycan degradation pathway

To identify taxa utilizing the glycan degradation pathway in each metagenome we selected contigs that encode enzymes of the pathway and quantified abundances of taxa representing these contigs. Overlay of the enzyme abundances across metagenomes with the relative abundances of taxa involved in glycan degradation (Fig. 4e) showed that major taxa degrading glycan are different between MA metagenomes of patients with small and large tumors. *Clostridiales* is more likely to encode enzymes of the glycan degradation in the LT-group, while *Bacteroidales* is the major taxon that encode the enzymes in ST-group. The result is consistent with the difference in taxonomic structure of MA metagenomes described in the previous paragraph. Further correlation analysis revealed a significant positive association of the glycan degradation pathway score with *Bacteroidales, Clostridiales, and* with non-classified taxa. A significant negative correlation was found with *Actinomycetales, Chlamydiales,* and *Tissierellales* (Additional File 1: Figure S17). In general, there was significant variation across taxa involved in glycan degradation. The variation was especially dramatic in MA metagenomes of patients with large tumors.

## Discussion

Herein, we have described significant differences in the gut metagenomes adjoining the mucus layer (MA metagenome) between cervical cancer patients with small, early-stage tumors and large, advanced stage tumors at the time of treatment. Specifically, MA metagenomes of patients with small tumors were enriched in molecular functions associated with biosynthesis and rapid proliferation. By contrast, MA metagenomes of patients with large tumors were enriched with functions associated with bacterial stress response and degradation of glycan. In addition, the utilization of ethanolamine and functions related to bacterial cells mobility were more abundant among young patients with large tumors and advanced stage (Fig. 1c). Activities of glycan degradation and of ornithine biosynthesis were also significantly associated with the advance tumor stage and the lymph node metastasis, while activity of the ribosome biogenesis was significantly associated with low stage tumors and better recurrence-free survival. This dominance may lead to significant changes in the metabolic environment of the intestine that directly impact tumor growth and progression.

It is possible that the tumor biology is influenced by differing molecular functions in MA metagenome. We have found that MA metagenomes include two distinct microbial consortia, which we refer to here as proliferating and mucus degrading (Fig. 4f). Both consortia co-exist, although patients with larger tumor size and advanced stage show an obvious dominance of the mucus degrading consortia over proliferating. The tumor promoting effects of the degradation and utilization of major degradation products, such as galactose and other pentoses [19], may help drive the tumor growth. Glycan degradation leads to production of metabolites with known tumor growth promoting effects and with resistance to radio- and chemotherapy, such as ornithine [52] and ceramides [53, 54]. The metabolites are produced as by-products of glycan degradation (Fig. 1e) and can be further metabolized either by bacteria or by host and give oncogenic effect after their metabolization. Ceramide itself, for example, is a powerful tumor suppressor, but products of its metabolism are potent tumor survival factors associated with resistance to cancer therapies [53]. Ornithine can be used by the consortium or by the host to synthesize the polyamines putrescine and spermidine (Additional File 1: Figure S8), which cause tumorigenic transformation and tumor progression [55]. If the gut metabolites cross the gut mucosa into blood through the mucus layer, they may promote systemic proliferation of tumor cells. In addition, the intensive ethanolamine utilization (Fig. 1e), which is especially pronounced in younger patients (Fig. 1c) with larger tumors, also suggests a more pathogenic environment in the intestine. The set of enzymes involved in the pathway are similar to the *eut* operon in *Salmonella enterica serovar Typhimurium* [56], a known gastrointestinal pathogen. Many other species that contain the *eut* genes are also pathogens [46], because ethanolamine is derived from the membrane phospholipid phosphatidylethanolamine, an important component of all bacterial and eukaryotic cells. The intensive utilization of ethanolamine may indicate a degradation of the colonic epithelium [46] that secretes peptides inhibiting bacterial penetration into the inner colonic mucus layer and blocking bacterial mobility [17, 57]. Indeed, the activity of pathways associated with mobility, such as chemotaxis and flagella assembly, are also enhanced in MA metagenomes of the LT-group, particularly in sub-cluster of younger patients (Fig. 1c). Conversely, the increased synthesis of peptidoglycan and lipopolysaccharides by the proliferating consortia may improve the intestinal microenvironment and the immune response in patients with smaller tumors [58]. The increased production of vitamins B7 (biotin) and B12 (cobalamin) may also be important for immune cell function and reduction of cellular oxidative stress [59–61]. Overall, these metabolites may have a tumor suppressive effect.

Based on the contrasting functions, tumor promoting versus suppressive, implemented by proliferating and mucus degrading microbial communities, we speculate that a disbalance in their activities may contribute to CC progression and to more aggressive tumor phenotypes. This study does not make clear what comes first, mucus degradation or tumor progression. It is likely that the processes are bidirectional and influence each other. Cervical cancer typically develops over years. Differences in the microbial flora of a large tumor may reflect changes in the tumor microenvironment directly affected by local tumor progression, or gut metagenome functions may allow increased tumor growth via systemic factors released into the bloodstream. Alternatively, tumor size may be a surrogate for other aggressive tumor biology, such as hypoxia or necrosis, which increase as tumors grow. Although this study associates large tumor size with functions representing degradation of mucus layer and degradation by-products with known tumor promoting effects, it doesn’t demonstrate that the association is causal and doesn’t provide direct evidence for linking the produced metabolites with tumor size. It is possible that tumor progression and associated changes of organismal processes may be primary and drive changes in the mucus layer microenvironment and in molecular functions of the MA community through production of cytokines and metabolites [17] or by changing the mucin glycans that are also important host signals selecting microorganisms and making them less pathogenic [23]. Indeed, the study reveals stress-associated changes in the mucus layer microenvironment that are indicated by reduced proliferation, and by increased activity of quorum sensing and sporulation in mucus degrading consortium. In addition, the taxonomic structure of the consortium is different between metagenomes in large- and in small-tumor groups. The difference, however, is pronounced only when functions and pathways are also considered, suggesting taxonomic comparison alone is not sufficient. Further studies using mouse or cell culture models are necessary to prove biological assumptions made in the paper and to decipher biological mechanisms underlying the discovered association between CC and mucus layer degradation. Sequencing and analysis of DNA from cervical swabs in parallel with the rectal swabs in CC patients would be also important to explore the mechanisms.

The association of CC progression with MA metagenome functions suggest a potential therapeutic intervention by shifting the balance in the metagenome from mucus degrading consortium to proliferating for suppression of the tumor growth and for improving the treatment outcome. The intervention, however, will require to know the optimal balance between the proliferating and degrading consortia because the latter is also a known component of the healthy gut and is important for renewal of the mucus layer.

Because microbial communities populated the mucus layer are not specific to CC, it is likely that similar associations between the tumor size and structure and function of MA metagenomes may be seen in other cancer types. In addition, shifting the balance between proliferating and mucus degrading consortia in the gut may directly affect cancer therapy and drug toxicity. The mucus layer plays a very important protective role in the intestine [62], therefore its unbalanced degradation can impair sensitivity to or ability to resist toxic effects of drugs. Recently, a growing body of evidence linked microorganisms to cancer therapy efficacy and toxicity [8–10]. Our previous study of metagenomes in fecal samples of melanoma patients with different response to anti-PD-1 therapy [10] reveals that degradation associated pathways are enriched in non-responders, while biosynthetic pathways are enriched in responders. We were not able to associate catabolic pathways with the mucus layer degradation in this study; however, we can speculate that the degradation can be responsible for the enrichment of catabolic processes in non-responding patients. The small number of samples and the use of fecal samples instead of rectal swabs might complicate identification of the mucus layer degradation in the study. It is likely that fecal samples are not optimal for the evaluation because they represent a different environment in the intestine. Further studies using WGS are necessary to compare fecal and swab samples from the same patient when mucus layer degradation is evaluated.

## Conclusions

This study shows that the mucus adjoining environment in the rectum of CC patients contains two microbial communities, proliferating and mucus degrading, with potential tumor suppressive and tumor-promoting functions, respectively. Theoretically, the disturbed balance between the communities could be corrected by diet, probiotics, antibiotics, or other interventions. Further mechanistic studies are very important to confirm associations identified in this study.

## Supplementary Information

**Additional file 1:** **Figure S1.** Workflow for quantification and analysis of known functions in WGS dataset. a Computational pipeline for assembly and annotation of each metagenome. b Computational workflow for merging of metagenomes into MFA table. c MFA table analysis. **Figure S2.** A toy example explaining a quantification of molecular functions using WGS dataset. The quantity of a molecular function in the metagenome is referred as Metagenome Function Abundance (MFA). The Figure shows 2 sequenced Metagenomes, A and B, populated by 18 and 19 bacterial cells respectively that belong to 4 different species: read, green yellow and blue colored shapes. Circles have Function1 (F1) in the genome, and ovals have Function 2 (F2). We propose to calculate MFA as the total number of DNA sequencing reads covering known functions in all bacterial cells. In the depicted example, each bacterial cell shown as an ovals has a gene that encode F1, but doesn’t have genes encoding F2. Vice versa, each bacterial cell shown as a circle has a gene that encode F2, but not F1. In both cases, ovals and circles, cells belong to 2 different species shown by color. For simplicity, we suggest that all genes in each cell is covered by 1 read after WGS. Therefore, MFA of F1 may be calculated just as number of circles in each metagenome, which is 4 for metagenome A and 16x for metagenome B, MFA ofF2 will be equal 14 and 4 respectively for A and B. The produced MFA values are integrated into MFA table where columns are metagenomes in the study and rows are unique known functions found in the metagenomes. The quantification is based on the following 2 conditions: (1) We ignore which of the species implement the function and calculate the Metagenome Function Abundance (MFA) as the total number of reads that cover the function in each cell of the metagenome, (2) If we don’t know what function the gene implement, we just ignore the gene. One important advantage of the quantification is that the MFA profile calculated this way for all unique function identified in the metagenome is like the profile of gene abundances calculated from RNA-seq data and can be analyzed in a similar way. **Figure S3.** KEGG pathways associated with quick proliferation of microbial cells are enriched “ST KO cluster” (Fig. 1c). a Ribosome biogenesis. b DNA repair. c Oxidative phosphorylation. KEGG orthologous groups found in the clusters are labeled by rose color. **Figure S4.** Quorum sensing was enriched in metagenomes of “LT KO cluster” (Fig. 1c). Total 19 KOs were annotated by the pathway in the cluster with the most complete known pathways similar to Staphylococcus aureus and Enterococcus faecalis. **Figure S5.** Overlap of KOs involved in galactose, fructose and mannose metabolism with KOs found in “LT KO cluster” (Fig. 1c). Overlapping KOs are colored in rose. **Figure S6.** Overlap of genes involved in pentose phosphate pathway in “LT KO cluster” (Fig. 1c). Overlapping genes are colored in rose. **Figure S7.** Overlap of genes involved in bacterial chemotaxis (a) and flagella assembly (b) with KOs found in “LT KO cluster” (Fig. 1c). Overlapping KO are colored in rose color. **Figure S8.** Overlap of enzymes involved in galactose, arginine and proline metabolism with differentially abundant KOs between LT- and ST-groups of metagenomes. (Fig. 2j). Overlapping KO are colored in rose color. Metabolic routs involved in production of glutamate from arginine and in putrescine and spermidine metabolism are framed in red. **Figure S9.** Overlap of enzymes involved in 2-oxocarboxylic acid metabolism with differentially abundant KOs between LT- and ST-groups of metagenomes. (Fig. 2j). Overlapping KO are colored in rose color and framed in red. They represent the complete set of enzymes involved production of ornithine from glutamate. **Figure S10.** Association of clinicopathological characteristics with the progression free survival probability. Categorization of patients into the low (blue color) and high (rose color) group in terms of the continues characteristics, such as Age, BMI and Tumor size, was done according to the maximally selected rank statistics as described in the Methods section. Characteristics significantly associated with the progression free survival according to log-rank test are in red color. **Figure S11.** Increased activity of Ribosome biogenesis pathway in metagenomes of patients with stage I/II tumors. **Figure S12.** Taxonomic structure of metagenomes in LT- and ST-groups at the Class level. **Figure S13.** Taxonomic structure of metagenomes in LT- and ST-groups at the Order level. **Figure S14.** Taxonomic structure of metagenomes in LT- and ST-groups at the Family level. **Figure S15.** Differentially abundant taxa between Large (L) and Small (S) tumor groups (a) and cladogram of the taxa (b) identified by LDA Effect Size (LEfSe) **Figure S16.** Relative abundance of selected taxa differentially abundant in ST-group (a) and in LT-group (b) according to LEfSe analysis. **Figure S17.** Pairwise Person correlation coefficients between the Glycan Degradation pathway score and the abundance profile of the taxa (order level) encoding enzymes of the pathway. Coefficients identified as significant (P<0.05) are labelled by asterisk. Color of the column indicate positive (red) and negative (blue) association between abundance of the order and the pathway score.

**Supplementary file 2:** **Table S1.** Software tools/packages used in the shotgun metagenome sequencing data analysis workflow. **Table S2.** Clinicopathological characteristics of the study cohort. **Table S3.** Functional richness of metagenomes identified in each patient of the study cohort. **Table S4.** Differentially abundant taxa identified between LT- and ST-groups by linear discriminant analysis (LDA) effect size (LEfSe) analysis.

## Data Availability

The datasets used and/or analyzed during the current study are available from the corresponding author on reasonable request. Raw sequencing reads generated by WGS are available in the BioProject database under accession PRJNA702617(https://www.ncbi.nlm.nih.gov/bioproject/PRJNA702617).

## Abbreviations

BMI: Body Mass Index
CAZyme: CArbohydrate-active enZymes
CC: Cervical Cancer
FIGO: The International Federation of Gynecology and Obstetrics
gDNA: Genomic bacterial DNA
GH: Glycoside hydrolases
GT: Glycosyl Transferases
HPV: Human PapillomaVirus
IRB: Institutional Review Board
KEGG: Kyoto Encyclopedia of Genes and Genomes
KO: KEGG Orthologous group
LBJ: Lyndon B. Johnson Clinic at Harris Health
LEfSe: Linear discriminant analysis Effect Size
LT: Large Tumors
MA: Mucus Associated
MDACC: M.D. Anderson Cancer Center
MFA: Metagenome Function Abundance table
OTU: Operating Taxonomic Unit
PTS: PhosphoTransferase System
RFS: Recurrence free survival
rRNA: Ribosomal RbonNucleic Acid
16Sv4: 16S rRNA marker gene
ST: Smaller Tumors
WGS: Whole Genome Shotgun
