# Supplementary material for: Metagenomes of rectal swabs in larger, advanced stage cervical cancers have enhanced mucus degrading functionalities and distinct taxonomic structure
Source: BMC Cancer. 2022 Sep 1;22:945. doi: 10.1186/s12885-022-09997-0 (PMC9438314; doi:10.1186/s12885-022-09997-0)
Supplement: Supplementary file 1 — Additional file 1: Figure S1. Workflow for quantification and analysis of known functions in WGS dataset. a Computational pipeline for assembly and annotation of each metagenome. b Computational workflow for merging of metagenomes into MFA table. c MFA table analysis. Figure S2. A toy example explaining a quantification of molecular functions using WGS dataset. The quantity of a molecular function in the metagenome is referred as Metagenome Function Abundance (MFA). The Figure shows 2 sequenced Metagenomes, A and B, populated by 18 and 19 bacterial cells respectively that belong to 4 different species: read, green yellow and blue colored shapes. Circles have Function1 (F1) in the genome, and ovals have Function 2 (F2). We propose to calculate MFA as the total number of DNA sequencing reads covering known functions in all bacterial cells. In the depicted example, each bacterial cell shown as an ovals has a gene that encode F1, but doesn’t have genes encoding F2. Vice versa, each bacterial cell shown as a circle has a gene that encode F2, but not F1. In both cases, ovals and circles, cells belong to 2 different species shown by color. For simplicity, we suggest that all genes in each cell is covered by 1 read after WGS. Therefore, MFA of F1 may be calculated just as number of circles in each metagenome, which is 4 for metagenome A and 16x for metagenome B, MFA ofF2 will be equal 14 and 4 respectively for A and B. The produced MFA values are integrated into MFA table where columns are metagenomes in the study and rows are unique known functions found in the metagenomes. The quantification is based on the following 2 conditions: (1) We ignore which of the species implement the function and calculate the Metagenome Function Abundance (MFA) as the total number of reads that cover the function in each cell of the metagenome, (2) If we don’t know what function the gene implement, we just ignore the gene. One important advantage of the quantification is that the MFA profile [file 12885_2022_9997_MOESM1_ESM.pdf]

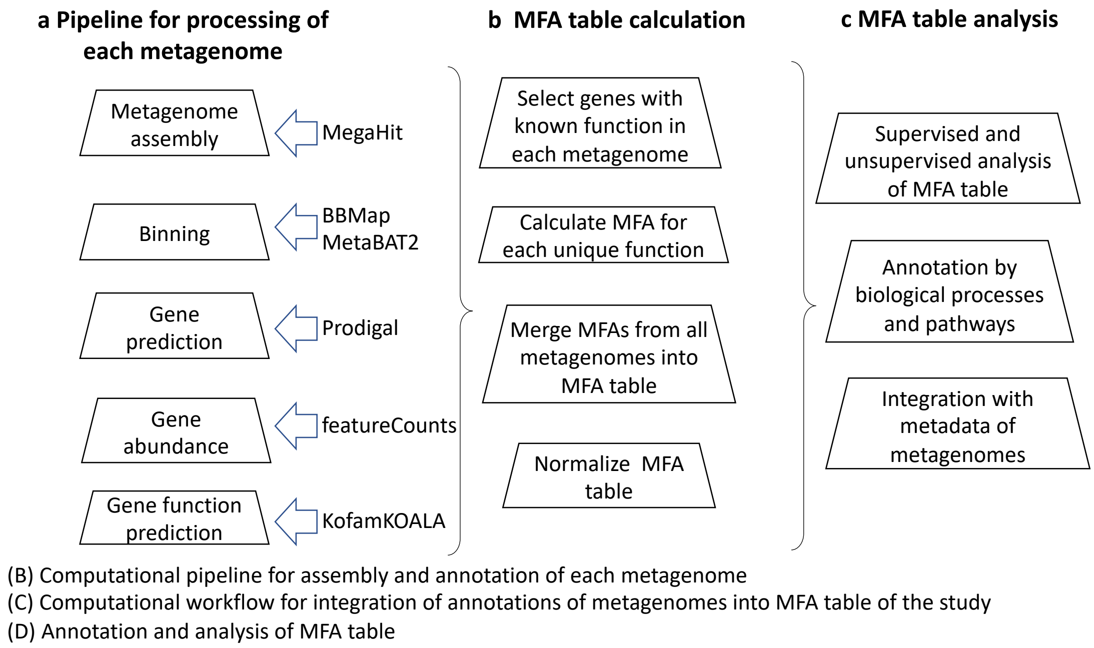


**Figure S1. Workflow for quantification and analysis of known functions in WGS dataset.** a Computational pipeline for assembly and annotation of each metagenome. b Computational workflow for merging of metagenomes into MFA table. c MFA table analysis.

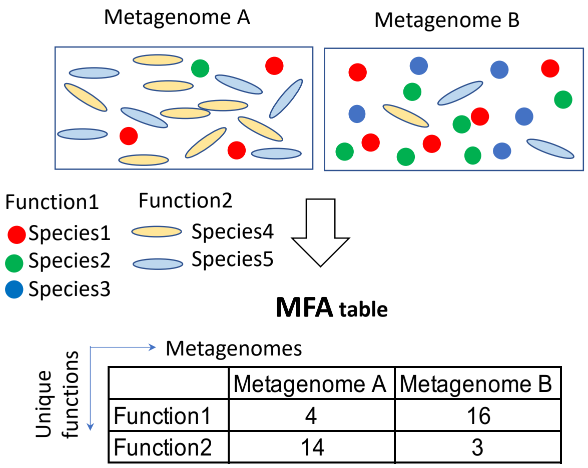


**Figure S2. A toy example explaining a quantification of molecular functions using WGS dataset.**

The quantity of a molecular function in the metagenome is referred as Metagenome Function Abundance (MFA). The Figure shows 2 sequenced Metagenomes, A and B, populated by 18 and 19 bacterial cells respectively that belong to 4 different species: read, green yellow and blue colored shapes. Circles have Function1 (F1) in the genome, and ovals have Function 2 (F2). We propose to calculate MFA as the total number of DNA sequencing reads covering known functions in all bacterial cells. In the depicted example, each bacterial cell shown as an ovals has a gene that encode F1, but doesn't have genes encoding F2. Vice versa, each bacterial cell shown as a circle has a gene that encode F2, but not F1. In both cases, ovals and circles, cells belong to 2 different species shown by color. For simplicity, we suggest that all genes in each cell is covered by 1 read after WGS. Therefore, MFA of F1 may be calculated just as number of circles in each metagenome, which is 4 for metagenome A and 16x for metagenome B, MFA ofF2 will be equal 14 and 4 respectively for A and B. The produced MFA values are integrated into MFA table where columns are metagenomes in the study and rows are unique known functions found in the metagenomes. The quantification is based on the following 2 conditions: (1) We ignore which of the species implement the function and calculate the Metagenome Function Abundance (MFA) as the total number of reads that cover the function in each cell of the metagenome, (2) If we don't know what function the gene implement, we just ignore the gene. One important advantage of the quantification is that the MFA profile calculated this way for all unique function identified in the metagenome is like the profile of gene abundances calculated from RNA-seq data and can be analyzed in a similar way.

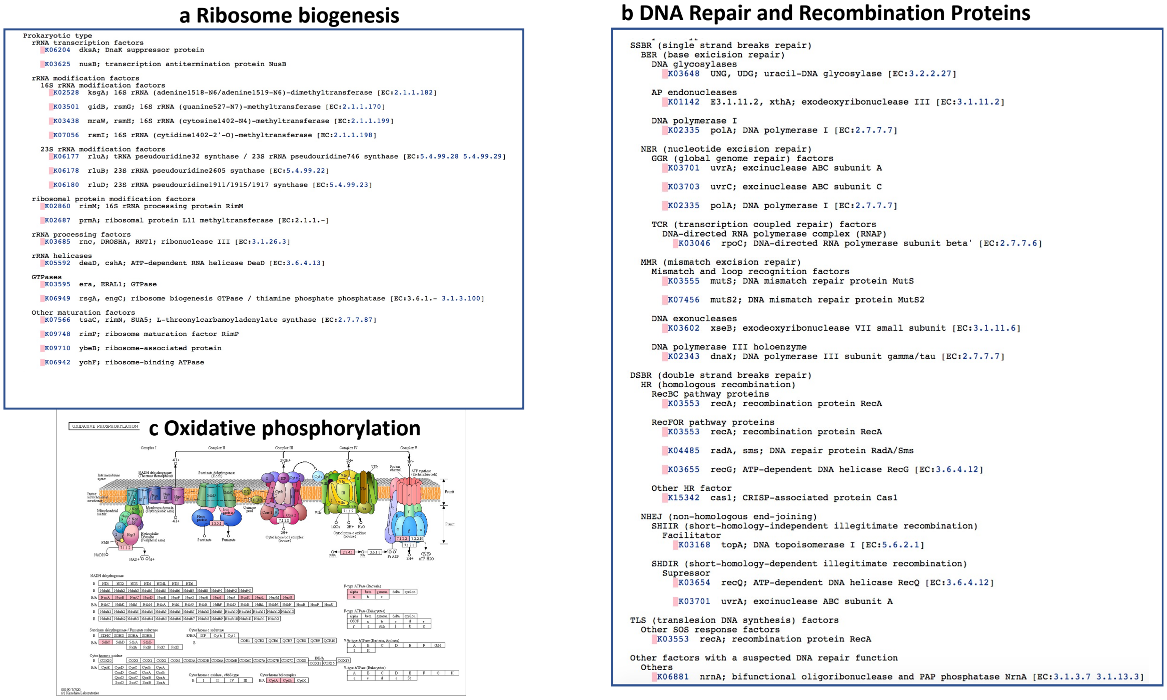


**Figure S3. KEGG pathways associated with quick proliferation of microbial cells are enriched "ST KO cluster" (Fig. 1c). a** Ribosome biogenesis. **b** DNA repair. **c** Oxidative phosphorylation. KEGG orthologous groups found in the clusters are labeled by rose color.

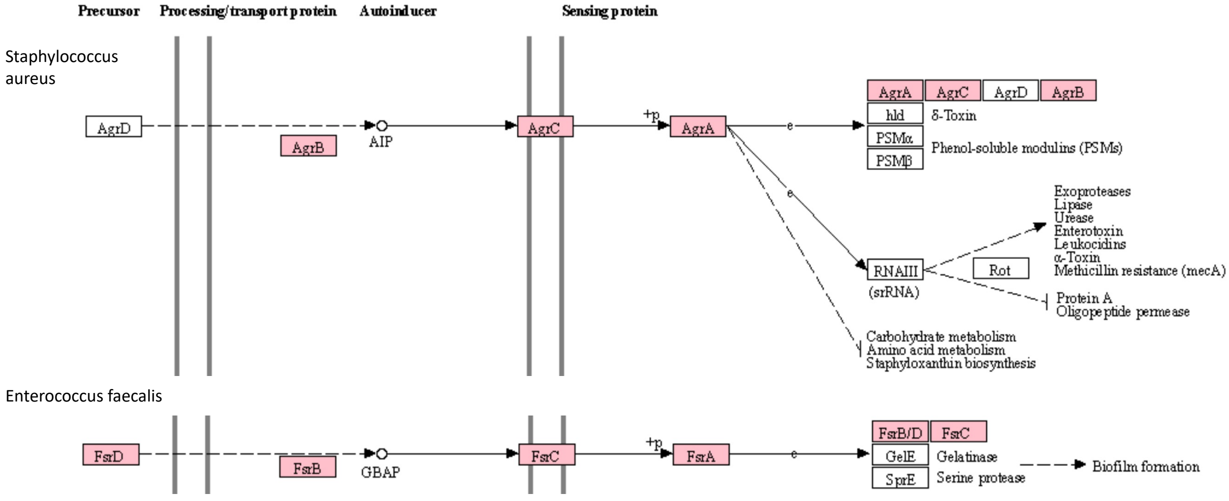


**Figure S4**. **Quorum sensing was enriched in metagenomes of** "LT KO cluster" (Fig. 1c). Total 19 KOs were annotated by the pathway in the cluster with the most complete known pathways like *Staphylococcus aureus* and *Enterococcus faecalis*

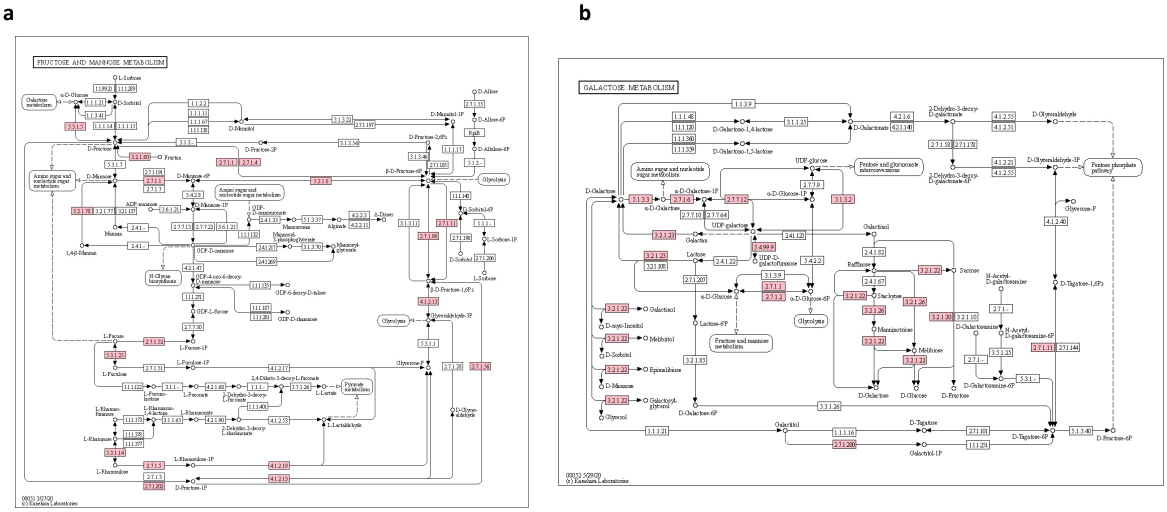


**Figure S5. Overlap of KOs involved in galactose, fructose and mannose metabolism with KOs found in** “LT KO cluster” (Fig. 1c). Overlapping KOs are colored in rose.

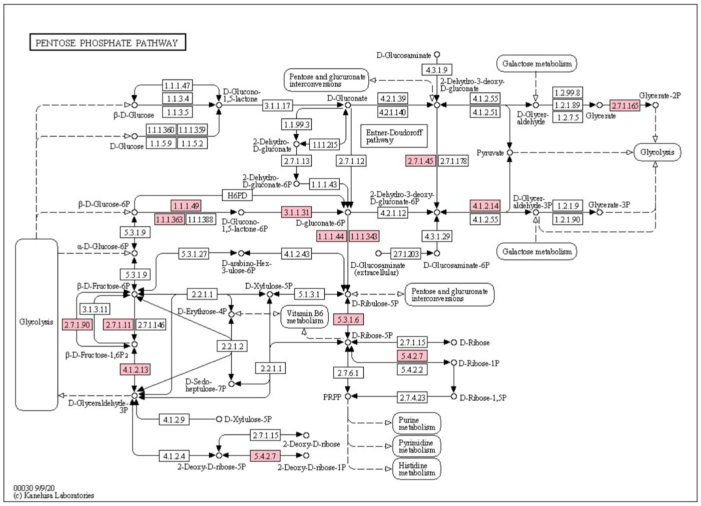


**Figure S6. Overlap of genes involved in pentose phosphate pathway in “LT KO cluster” (Fig. 1c).** Overlapping genes are colored in rose.

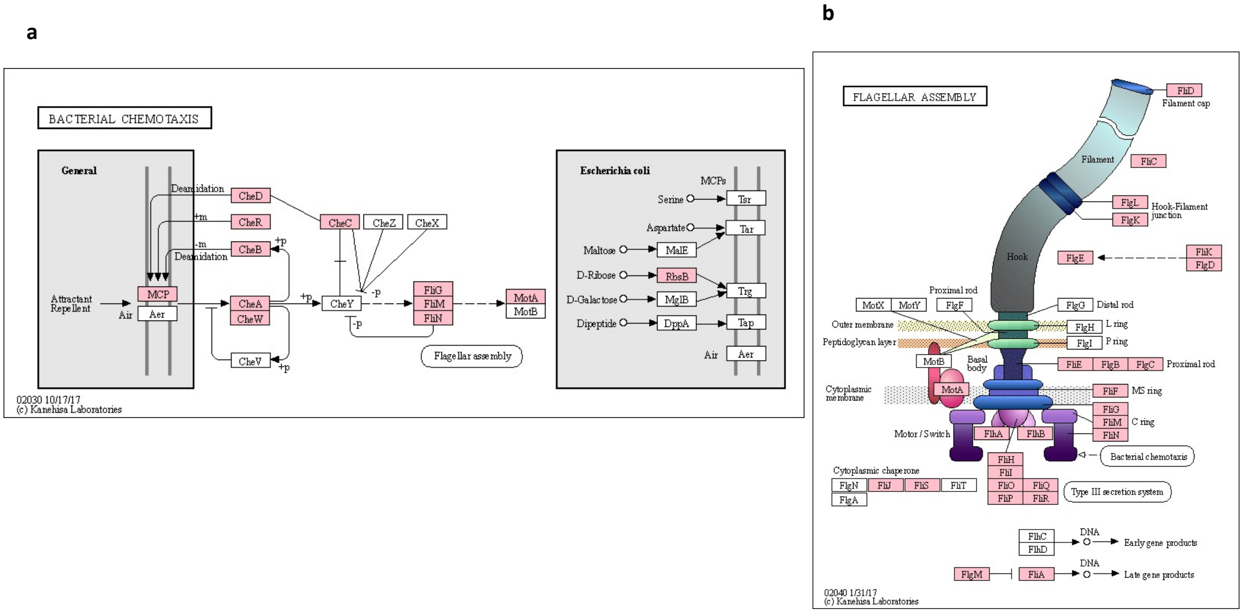


**Figure S7. Overlap of genes involved in bacterial chemotaxis (a) and flagella assembly (b) with KOs found in "LT KO cluster" (Fig. 1c).** Overlapping KO are colored in rose color.

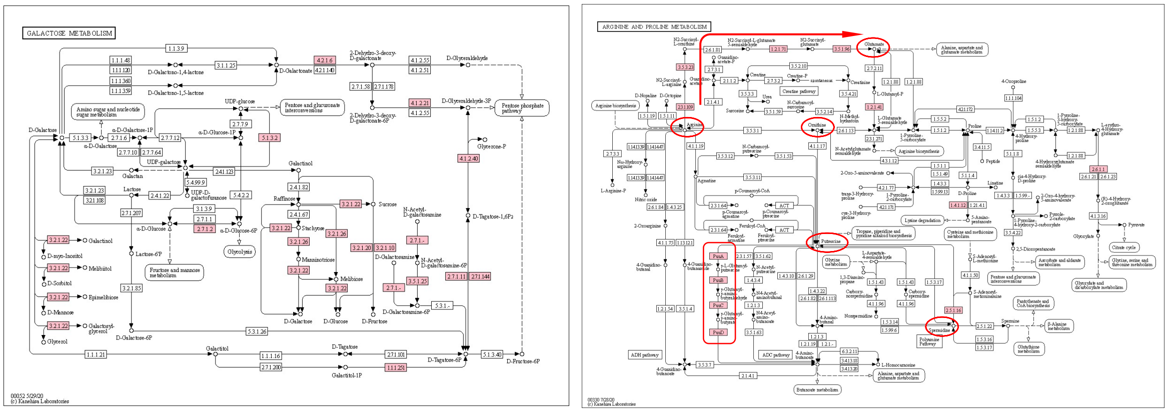


**Figure S8. Overlap of enzymes involved in galactose, arginine and proline metabolism with differentially abundant KOs between** LT- and ST-groups of metagenomes. (Fig. 2j). Overlapping KO are colored in rose color. Metabolic routs involved in production of glutamate from arginine and in putrescine and spermidine metabolism are framed in red.

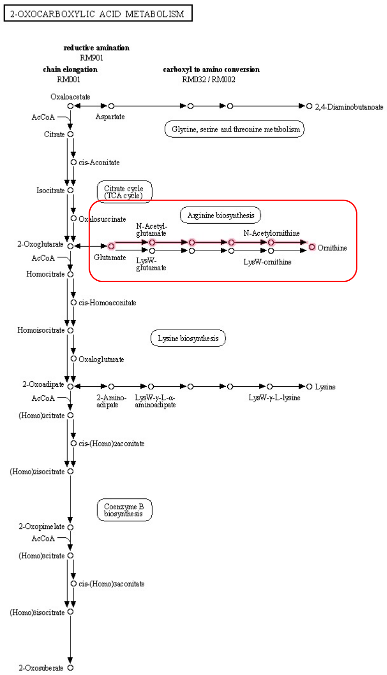


**Figure S9. Overlap of enzymes involved in 2-oxocarboxylic acid metabolism with differentially abundant KOs between LT- and ST-groups of metagenomes. (Fig. 2j).** Overlapping KO are colored in rose color and framed in red. They represent the complete set of enzymes involved production of ornithine from glutamate.

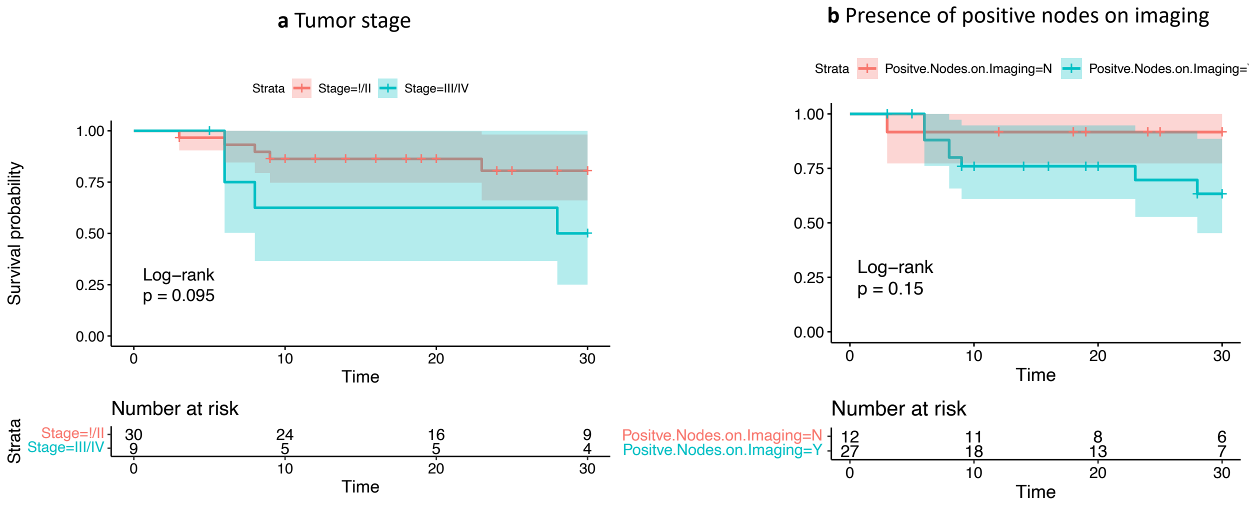


**Figure S10. Association of clinicopathological characteristics with the progression free survival probability.** Categorization of patients into the low (blue color) and high (rose color) group in terms of the continues characteristics, such as Age, BMI and Tumor size, was done according to the maximally selected rank statistics as described in the Methods section. Characteristics significantly associated with the progression free survival according to log-rank test are in red color.

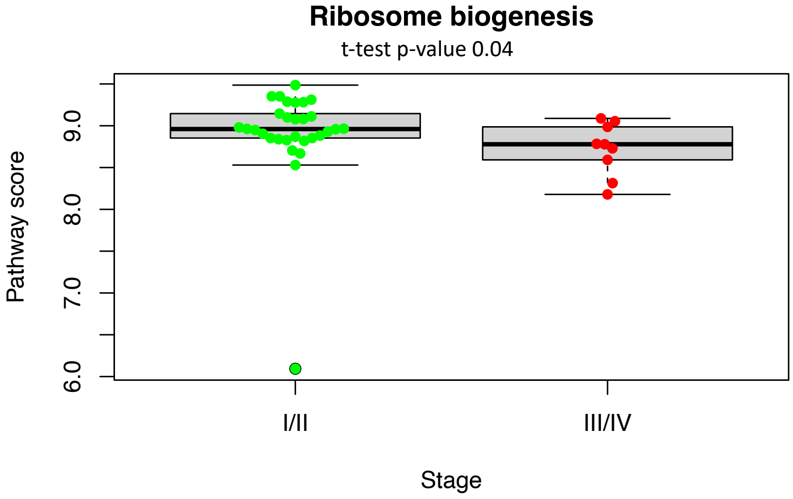


**Figure S11. Increased activity of Ribosome biogenesis pathway in metagenomes of patients with stage I/II tumors.**

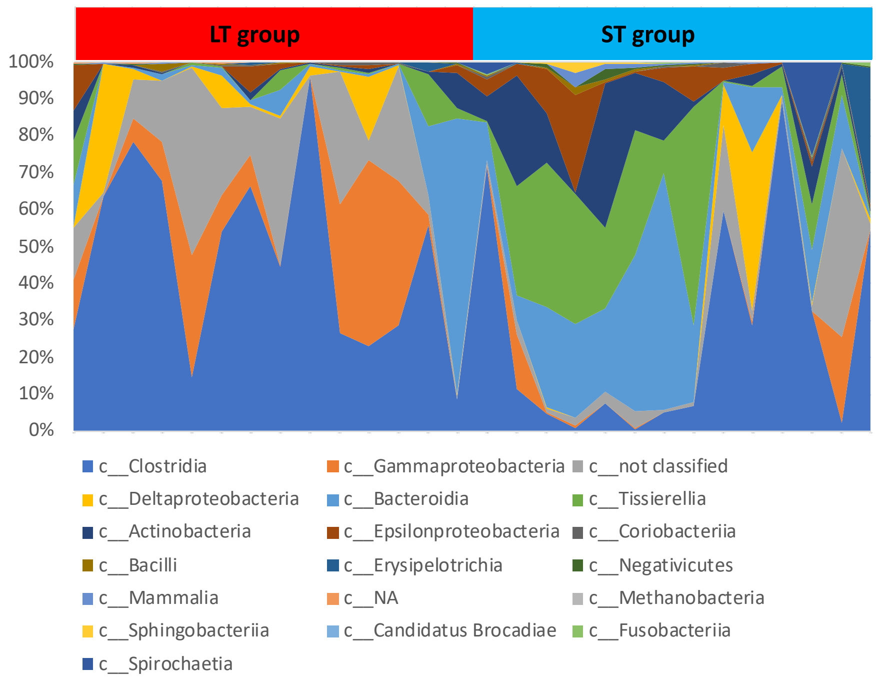


**Figure S12. Taxonomic structure of metagenomes in LT- and ST_groups at the *Class* level.**

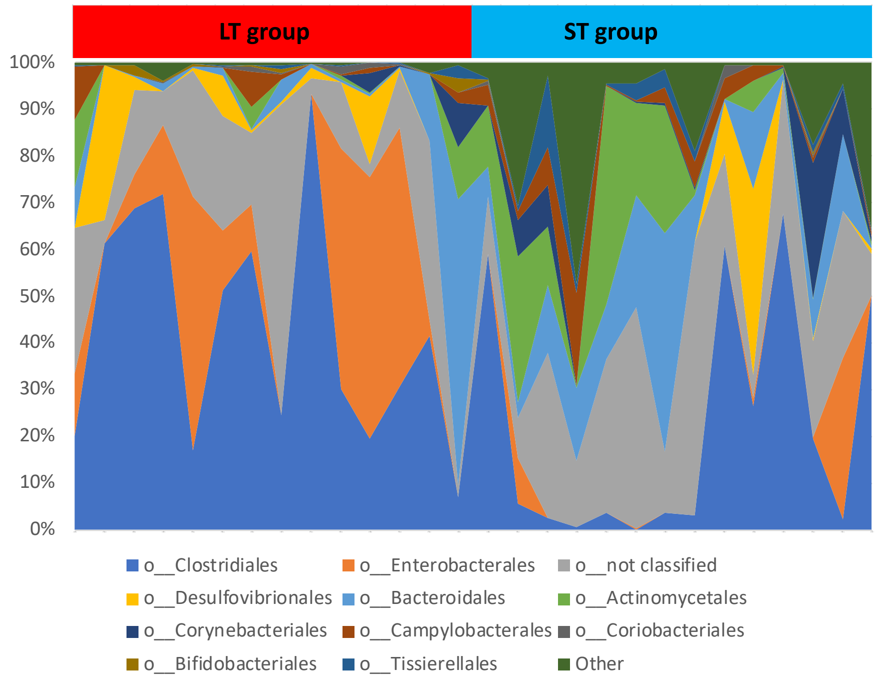


**Figure S13. Taxonomic structure of metagenomes in LT- and ST_groups at the *Order* level.**

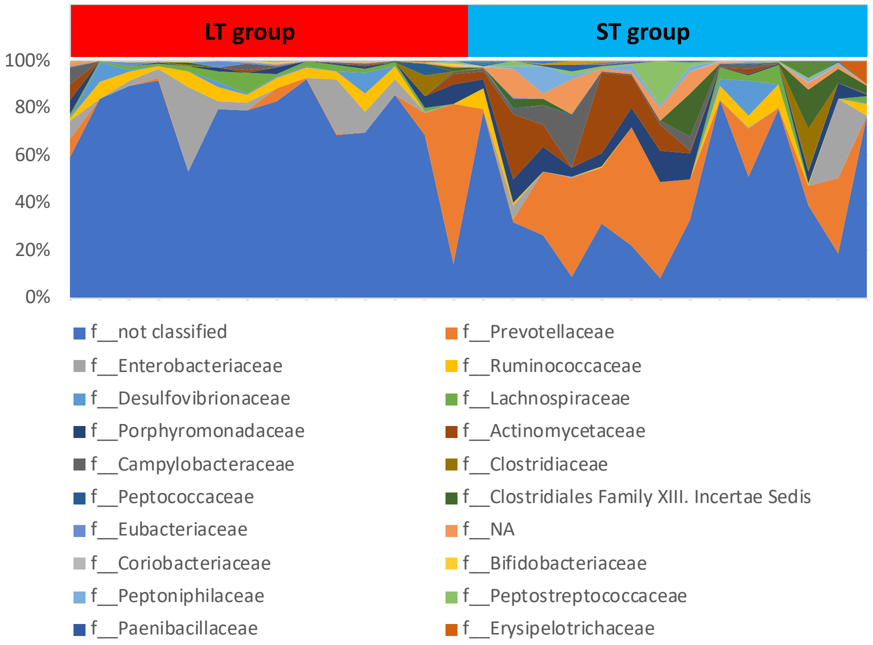


**Figure S14. Taxonomic structure of metagenomes in LT- and ST_groups at the *Family* level.**

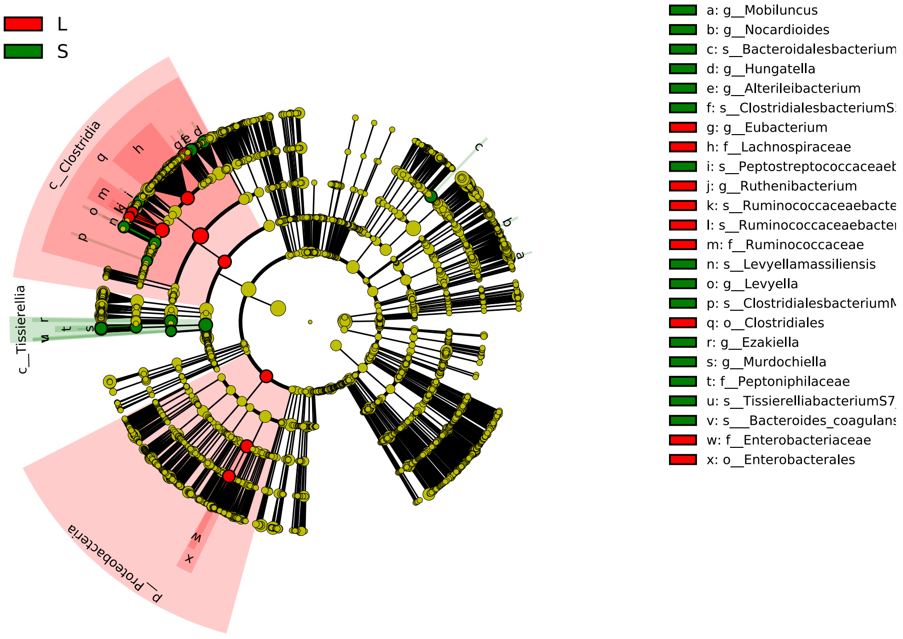


**Figure S15. Cladogram of differentially abundant taxa between LT- and ST-groups.**

The groups are labelled by L and S respectively.

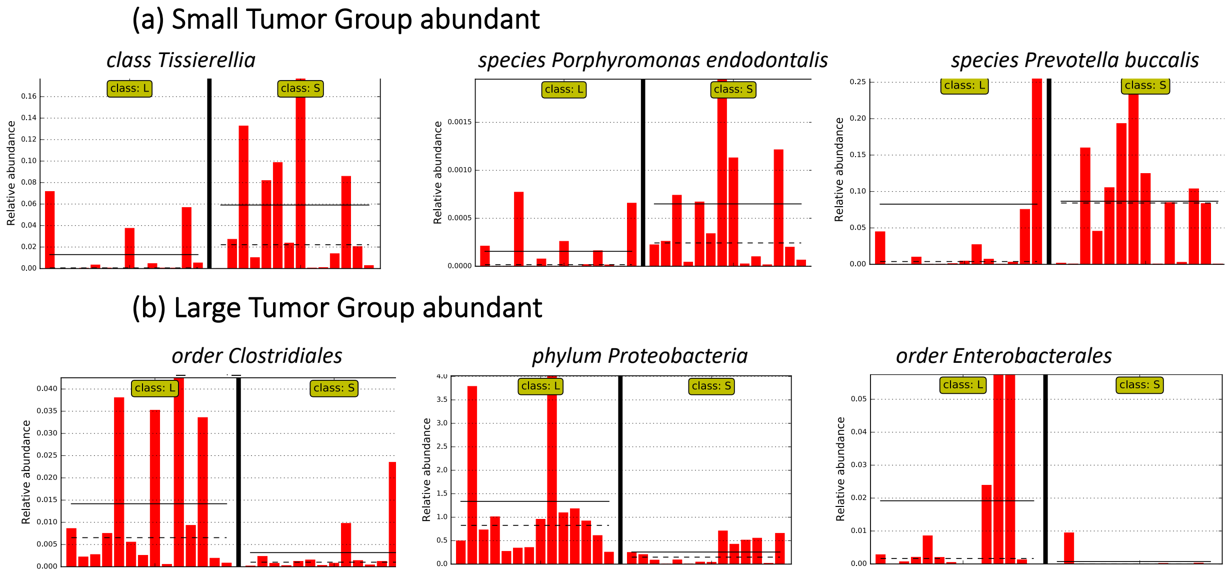


**Figure S16. Relative abundance of selected taxa differentially abundant in ST-group (a) and in LT-group (b) according to LEfSe analysis.**

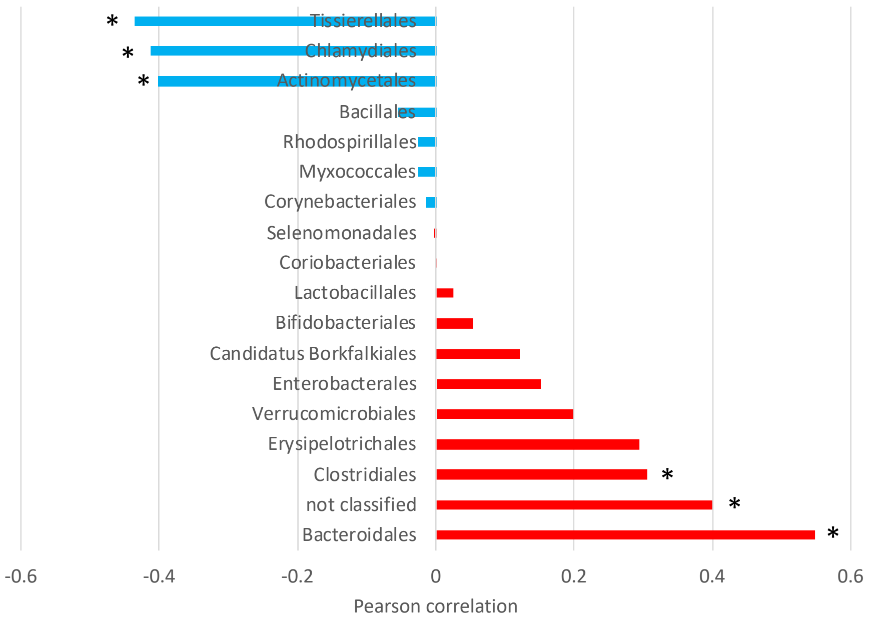


**Figure S17. Pairwise Person correlation coefficients between the Glycan Degradation pathway score and the abundance profile of the taxa (order level) encoding enzymes of the pathway.** Coefficients identified as significant ($P<0.05$) are labelled by asterisk. Color of the column indicate positive (red) and negative (blue) association between abundance of the order and the pathway score.
